# Supplementary material for: Microstructure Evolution and Its Correlation with Performance in Nitrogen-Containing Porous Carbon Prepared by Polypyrrole Carbonization: Insights from Hybrid Calculations
Source: Materials (Basel). 2022 May 22;15(10):3705. doi: 10.3390/ma15103705 (PMC9147853; doi:10.3390/ma15103705)
Supplement: Supplementary file 1 [file materials-15-03705-s001.zip › materials-1702764-supplementary.pdf]

Supporting Information

Microstructure Evolution and Its Correlation with Performance in Nitrogen-containing Porous Carbon Prepared by Polypyrrole Carbonization：Insights from Hybrid Calculations

ShanShan Li[1,2], Fang Bian[2], XinGe Wu[1], LeLe Sun[3], HongWei Yang[4], XiangYing Meng[1,5*], and GaoWu Qin[2,5]

[1]*College of Sciences, Northeastern University, Shenyang 110819, China*

[2]*Key Laboratory for Anisotropy and Texture of Materials (MoE), School of Materials Science and Engineering, Northeastern University, Shenyang 110819, China*

[3]*College of Information Science and Engineering, Northeastern University, Shenyang 110819, China*

[4]*State Key Laboratory of Advanced Technologies for Comprehensive Utilization of Platinum Metals, Kunming Precious Metals New Materials Technology Co., Ltd., Kunming 650106, China*

[5]*The State Key Laboratory of Rolling and Automation, Northeastern University, Shenyang 110819, China*

**Corresponding author:* Xiangying Meng

E-mail: x_y_meng@mail.neu.edu.cn

1. Molecular models

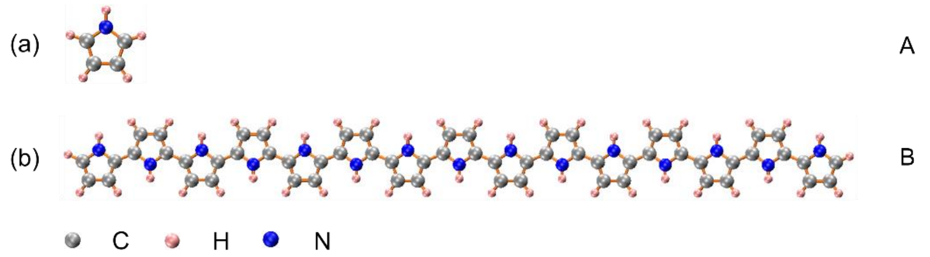

Figure S1. (a) The pyrrole molecule is characterized by a five-membered (5-M) ring composed of four carbon atoms and one nitrogen atom with a formula of C_4H_5N. (b) Polypyrrole (PPy) is a conductive organic polymer with a pyrrole chain.

2. Tissue correlation with electronic conductance

2.1 Computational model and determination of the optimum distance between buffer and scattering

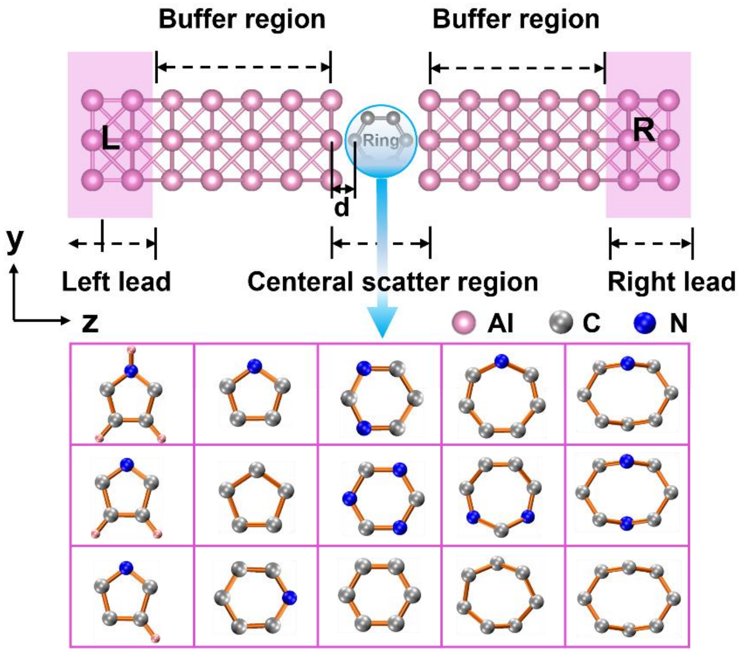

Figure S2. Schematic transport model for the calculations of ring conductance.

A two-probe transport model was constructed to calculate the electronic conductance, whose schematic diagram is illustrated in Supplementary Figure S2. A slab containing the two layers of Al atoms was used in the electrode region as the electrode's repetitive unit. The Al slab was extended along the *z*-direction to provide the bias and to collect the current. Different rings acted as the central scattering region.

The buffer region consisted of five layers of Al atoms in the *z*-direction to isolate possible interactions between the electrode and the central scattering region. The energy curve between the ring and the buffer region with distance was shown in Supplementary Figure S3. The optimized distance between the ring and the buffer region was 1.2 Å based on the results of the DFT total energy calculations.

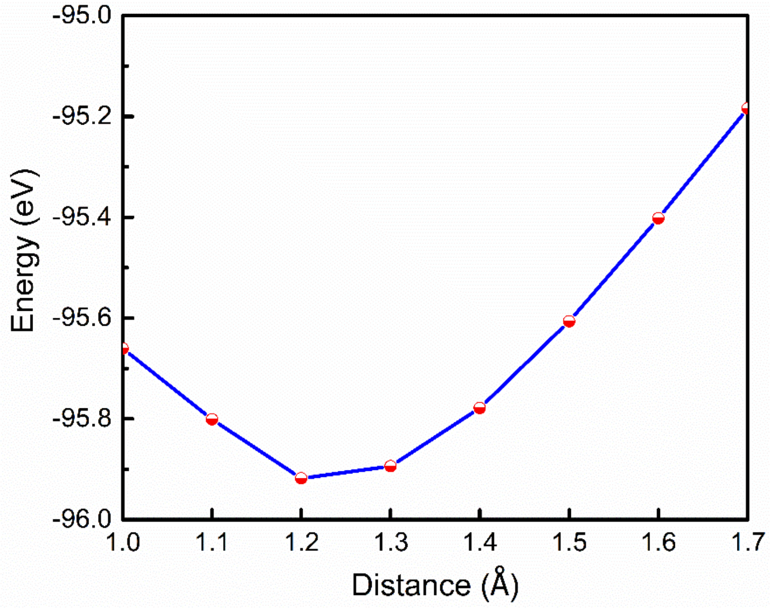

Figure S3. The curve of energy between the carbon ring and buffer zone with distance.

2.3 Machine learning details

The XGBoost algorithm was used to analyze the weights of the effect of ring structures on conductance. Supplementary Figure S4 shows that the top four features affecting conductance are 5-M ring, 5-MN$_1$ ring, 6-M ring, and 6-MN$_1$ ring, whose total weights are close to 70%.

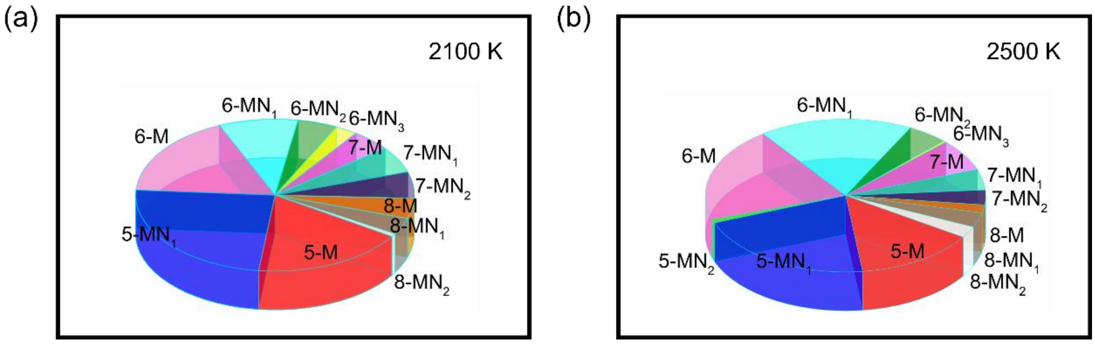

Figure S4. The weight of the effect of the ring structure on the conductance at different temperatures.

The top four features were used to construct the initial feature space Φ_0. The SISSO approach was executed to mine the functional relationship between the target property and features. After two iterations, the features most closely related to the target property were filtered through sure independence screening (SIS). The sparsifying operator (SO) fitted several structure-activity relationship models with the specific root mean square error (RMSE). The formulas are shown in Supplementary Table S1.

Table S1. The conductance formula and root mean square error (RMSE) were obtained by machine learning training.

2100 K		2500 K	
Formula	RMSE	Formula	RMSE
$0.5861 * \sqrt[3]{N_{6-M}/N_{5-MN_1}} + 0.9084$	0.035	$0.4326 * \sqrt[3]{N_{6-M}/N_{5-MN_1}} + 1.0453$	0.039
$0.2392 * \sqrt[3]{N_{6-M}} + 0.858$	0.036	$-0.0092 * (N_{5-MN_1} - N_{6-M}) + 1.4922$	0.043
$-0.0088 * \lvert N_{6-M} - N_{5-MN_1} \rvert + 1.3633$	0.036	$0.0105 * (\lvert N_{6-M} - N_{6-MN_1} \rvert - N_{5-MN_1}) + 1.6203$	0.044
$0.0709 * \sqrt[3]{(N_{6-M})^2} + 1.008$	0.036	$-0.0099 * (N_{5-MN_1} + N_{6-MN_1} - N_{6-M}) + 1.6237$	0.046
$0.2465 * \sqrt[3]{N_5 + N_6} + 0.7671$	0.038	$0.4975 * \sqrt[9]{N_{6-M}} + 0.9092$	0.055
……	……	……	……

Table S2 is the training set used in machine learning for conductance training.

Table S2. Machine learning training set for 2100 K. The unit of pore size is Å, the unit of time is ns, and the unit of conductance is $2e^2/h$.

Time (ns)	*5-M*	*5-M N₁*	5-M N_2	*6-M*	*6-M N₁*	6-M N_2	6-M N_3	7-M	7-M N_1	7-M N_2	8-M	8-M N_1	8-M N_2	Conductance	Pore size
0	0	60	0	0	0	0	0	0	0	0	0	0	0	0.9319	2.52933
0.1	1	42	0	0	2	0	0	0	0	0	0	0	0	0.962336	2.32047
0.2	2	33	0	2	5	0	0	0	0	0	0	0	0	1.057433	2.27440
0.3	2	34	0	2	4	0	0	0	0	0	0	0	0	1.049462	2.56682
0.4	1	27	0	2	2	0	0	0	0	0	1	0	0	1.046824	2.42854
0.5	2	24	0	2	4	0	0	1	1	1	0	0	1	1.151617	2.41955
0.6	1	27	0	3	5	0	0	1	0	0	0	0	0	1.109741	2.83716
0.7	3	22	0	2	3	0	0	2	0	0	1	0	0	1.154455	2.77166
0.8	3	23	0	3	5	0	0	3	0	1	2	0	0	1.208528	2.89142
0.9	2	21	0	5	6	1	0	3	3	0	2	0	0	1.306893	3.09302
1	3	23	0	6	5	3	0	3	2	0	1	0	0	1.30623	2.69818
1.1	5	22	0	7	4	2	0	3	2	1	0	0	1	1.347445	2.71236
1.2	7	26	0	9	6	4	0	3	1	1	1	0	0	1.34241	2.89992
1.3	5	23	0	9	5	4	0	3	2	1	1	0	1	1.363743	2.98615
1.4	5	17	0	10	8	4	0	3	0	0	3	0	1	1.376996	2.98163

1.5	4	19	0	12	6	4	0	4	1	0	1	0	0	1.423992	3.29306
1.6	4	20	0	13	5	6	0	5	2	0	1	0	0	1.449354	3.09673
1.7	4	21	0	16	7	4	0	4	3	0	1	1	0	1.460582	2.85387
1.8	3	22	0	15	10	3	0	4	3	2	0	1	0	1.445435	2.68586
1.9	6	22	0	16	6	1	0	4	4	3	0	1	0	1.490267	2.96461
2	6	25	0	17	8	3	0	5	4	2	1	1	0	1.463721	3.15316
2.1	6	24	0	17	8	3	0	5	3	2	0	0	0	1.473399	2.97230
2.2	6	26	0	14	9	3	0	4	3	2	1	0	1	1.413686	3.31595
2.3	7	23	0	14	10	3	2	4	3	1	0	0	0	1.45327	3.17139
2.4	8	24	0	14	10	3	1	4	3	1	0	0	0	1.442916	2.97921
2.5	7	22	0	15	11	2	0	4	3	1	0	0	0	1.456282	2.97931
2.6	7	23	0	14	12	3	1	4	3	1	0	1	0	1.436057	3.13909
2.7	7	22	0	16	11	3	1	3	3	1	1	2	0	1.446679	3.41034
2.8	7	23	0	16	10	4	1	4	3	1	1	1	0	1.453811	2.92003
2.9	8	25	0	16	9	3	1	5	3	1	1	1	0	1.449897	2.91630
3	7	23	0	17	9	3	1	5	2	2	2	1	0	1.460788	3.05207
3.1	7	21	0	16	13	4	1	4	4	1	1	1	0	1.469389	3.10869
3.2	6	22	0	19	14	4	1	5	2	1	1	1	0	1.467747	4.35485
3.3	7	22	0	18	13	4	1	5	3	1	0	1	0	1.4778	3.31321
3.4	7	21	0	17	14	4	1	5	4	1	0	2	1	1.470743	3.46645
3.5	7	19	0	18	15	5	1	6	3	1	0	1	0	1.498166	3.52099
3.6	7	20	0	18	16	4	1	4	4	1	0	0	1	1.487361	3.23642
3.7	7	18	0	16	16	4	1	5	4	2	0	1	1	1.489396	3.57033
3.8	7	18	0	17	15	4	0	5	2	2	0	1	0	1.485035	3.77894
3.9	7	18	0	17	15	4	1	5	4	2	0	2	1	1.493014	3.81399
4	7	16	0	17	15	4	1	5	4	1	0	1	1	1.511867	3.41840
4.1	6	18	0	15	18	4	1	5	3	1	0	2	1	1.455618	3.00244
4.2	6	16	0	19	16	6	1	4	3	1	1	1	1	1.502052	4.1836
4.3	6	18	0	18	17	5	1	6	3	1	1	2	0	1.487008	3.39235
4.4	7	19	0	18	17	5	1	5	4	1	1	2	0	1.485231	3.64132
4.5	8	16	0	19	15	6	1	5	4	2	0	1	0	1.534992	3.60874
4.6	7	15	0	19	19	5	1	6	4	1	1	1	0	1.525263	3.55722
4.7	8	19	1	19	20	6	1	6	2	1	1	1	0	1.474735	3.70075
4.8	8	18	1	19	19	6	1	4	3	1	3	2	0	1.469346	3.61782
4.9	6	13	1	19	19	6	1	6	2	1	1	1	0	1.516192	3.99139
5	7	16	1	19	16	6	2	7	3	1	1	1	0	1.52027	4.31027
5.1	7	17	1	20	18	6	2	7	3	1	1	1	0	1.51331	3.95478
5.2	7	17	1	20	18	7	1	6	3	1	1	2	0	1.498935	3.59854
5.3	7	15	1	19	17	7	1	6	3	1	1	2	0	1.509361	4.14750
5.4	6	15	1	21	20	7	1	7	2	2	1	1	0	1.517406	4.61033
5.5	6	20	1	19	17	6	1	4	3	1	2	0	0	1.472558	4.89160
5.6	7	16	1	20	19	7	1	7	3	2	1	1	0	1.515725	3.62350
5.7	6	18	1	20	20	7	1	6	3	2	1	2	0	1.488446	4.26287
5.8	5	16	1	18	19	7	1	6	3	2	1	1	0	1.495878	3.89357

5.9	5	17	1	18	20	6	1	5	3	3	1	1	0	1.485578	4.39990
6	6	17	1	21	21	7	1	4	3	2	2	1	0	1.492283	4.12972

3. Details of PSD analysis and tissue correlation mining

By employing a machine learning strategy similar to the one used in the conductance study, we found that the top four features affecting pore size were 5-M ring, 5-MN_1 ring, 6-M ring, and 6-MN_1 ring, total weights close to 70%, as shown in Supplementary Figure S5.

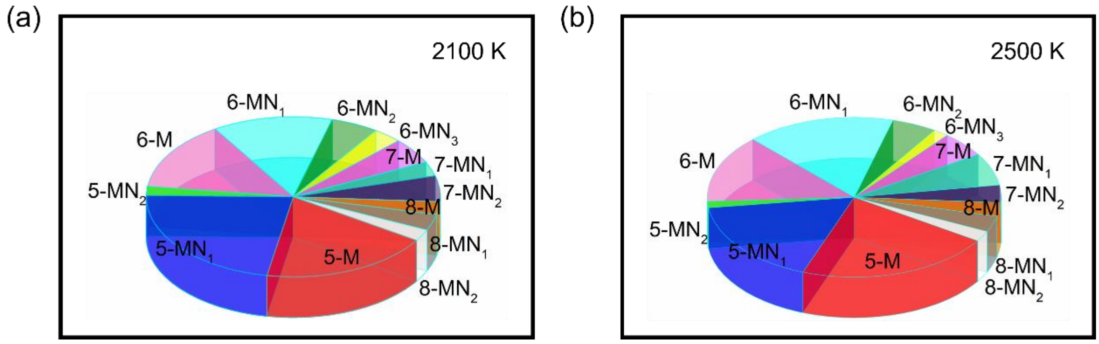

Figure S5. The weight of the effect of the ring structure on the pore size at different temperatures.

The PPy systems were equilibrated at 300 K for 100 ps, and the temperature and energy changes with time verified the equilibrium states, as shown in Supplementary Figure S6.

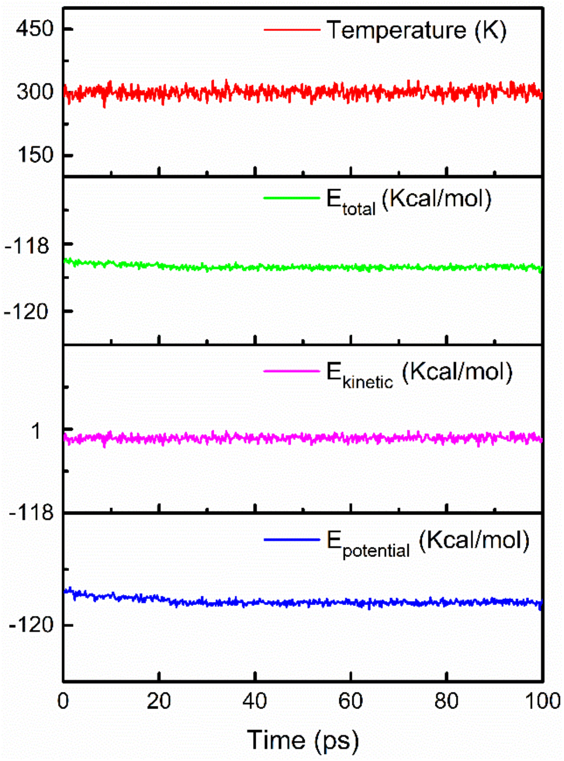

Figure S6. Temperature and energy change with time during constant temperature simulation at 300 K.

In supplementary Figure S7, we plotted the variation of PSD with time in the PPy conversion products at 2100 K and 2500 K, respectively. We found that the system's pore size increased with the reaction's progress at 2100 K, while at 2500 K, the pore size first increased, and when the graphite-like structure appeared, the pore size was slightly reduced. The final reduced pore size at 2500 K was similar to that at a lower temperature (2100 K).

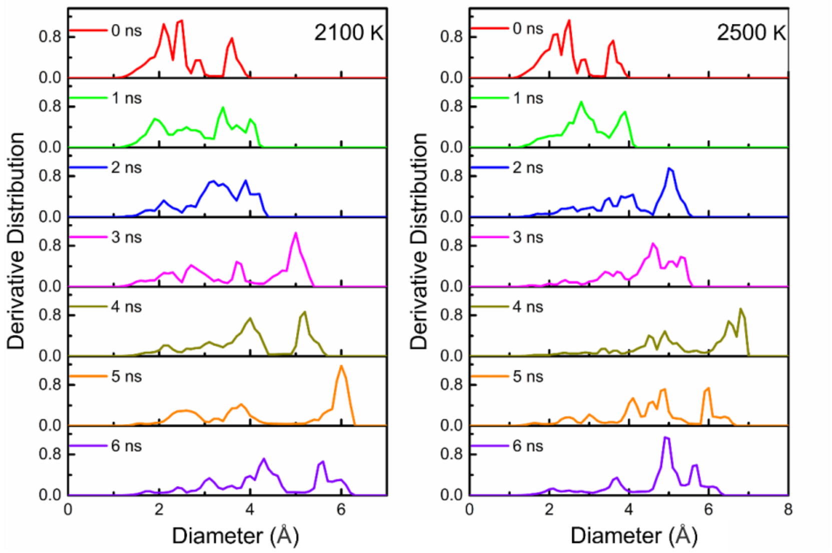

Figure S7. The pore size distribution (PSD) diagrams with time in the PPy conversion

products at 2100 K and 2500 K, respectively. The results were obtained by using a probe with a diameter of 0.5 Å.

Several relationship models of structures and pore size were fitted by the sparsifying operator (SO) with the specific root mean square error (RMSE). The formulas are shown in Supplementary Table S3.

Table S3. Machine learning training obtained the pore size formula.

2100 K		2500 K	
Formula	RMSE	Formula	RMSE
$0.0037 * (\mathrm{N}_{6-\mathrm{M}} * \mathrm{N}_{6-\mathrm{MN}_1} - \mathrm{N}_{5-\mathrm{MN}_1}) + 2.6928$	0.307	$0.0028 * (\mathrm{N}_{6-M} * \mathrm{N}_{6-\mathrm{MN}_1} - \mathrm{N}_{5-M\mathrm{N}_1}) + 3.0564$	0.425
$0.0002 * \mathrm{N}_{6-\mathrm{MN}_1} * (\mathrm{N}_{6-\mathrm{M}})^2 + 2.6847$	0.308	$0.0045 * (\mathrm{N}_{5-\mathrm{MN}_1} * (\mathrm{N}_{5-M} + \mathrm{N}_{6-M\mathrm{N}_1}) + 2.8318$	0.426
$0.0037 * (\mathrm{N}_{6-M} + \mathrm{N}_{6-M} * \mathrm{N}_{6-\mathrm{MN}_1}) + 2.5682$	0.31	$0.0029 * (\mathrm{N}_{6-M} * \mathrm{N}_{6-\mathrm{MN}_1} - \mathrm{N}_{6-M\mathrm{N}_1}) + 3.0389$	0.426
$0.0038 * (\mathrm{N}_{6-M} * \mathrm{N}_{6-\mathrm{MN}_1} - \mathrm{N}_{5-M}) + 2.6035$	0.31	$0.0028 * (\mathrm{N}_{6-M} * \mathrm{N}_{6-\mathrm{MN}_1}) + 3.0268$	0.426
$0.004 * (\mathrm{N}_{6-M} * \mathrm{N}_{6-\mathrm{MN}_1} - \mathrm{N}_{6-M\mathrm{N}_1}) + 2.603$	0.31	$0.0027 * (\mathrm{N}_{6-\mathrm{MN}_1} + \mathrm{N}_{6-M} * \mathrm{N}_{6-M\mathrm{N}_1}) + 3.0159$	0.426
......	......	......	......

Table S4 is the training set used in machine learning for pore size training.

Table S4. Machine learning training set for 2500 K. The unit of pore size is Å, the unit of time is ns, and the unit of conductance is $2e^2/h$.

Time (ns)	*5-M*	*5-M N_1*	5-M N_2	*6-M*	*6-M N_1*	6-M N_2	6-MN 3	7-M	7-M N_1	7-M N_2	8-M	8-M N_1	8-M N_2	Conductance	Pore size
0	0	60	0	0	0	0	0	0	0	0	0	0	0	0.9319	2.52288
0.1	3	17	0	4	4	0	0	1	0	0	0	0	0	1.233434	2.43786
0.2	7	6	0	8	1	0	0	1	0	0	0	1	0	1.557108	2.66337
0.3	9	11	0	13	6	4	1	2	4	0	0	0	0	1.573238	3.61751
0.4	9	6	0	10	15	3	1	2	3	0	1	1	0	1.527643	3.07865
0.5	8	4	0	11	11	2	3	1	2	0	1	1	0	1.618865	3.48206
0.6	6	9	0	17	14	2	1	4	3	1	0	0	0	1.561872	3.11016
0.7	10	7	0	18	11	3	1	2	2	0	2	1	0	1.591289	3.05258
0.8	7	7	0	15	13	3	0	4	5	0	0	0	0	1.613459	3.04509
0.9	11	5	0	16	14	2	1	2	4	0	2	0	0	1.613667	3.89562
1	10	9	0	20	11	2	1	3	5	0	4	0	1	1.60092	3.25844
1.1	11	10	0	16	10	4	1	2	5	0	2	0	0	1.5832	3.14559
1.2	10	10	0	19	6	3	1	4	7	1	1	0	0	1.661526	3.17095
1.3	9	7	0	22	9	3	1	7	4	1	1	0	0	1.682439	3.58964
1.4	11	7	0	22	11	2	1	8	3	1	2	3	1	1.623807	3.51276

1.5	14	7	0	24	13	3	1	7	6	1	2	0	0	1.671	3.64310
1.6	12	3	0	23	11	3	1	11	7	0	1	0	0	1.765421	3.44230
1.7	10	7	0	24	12	2	1	7	4	0	1	1	0	1.666259	3.33059
1.8	11	7	0	21	7	3	0	7	9	1	1	0	0	1.71796	3.72080
1.9	9	6	0	23	7	4	1	7	7	0	1	0	0	1.727138	3.24942
2	10	8	0	26	9	4	1	7	6	0	2	1	0	1.681545	3.61994
2.1	10	12	0	26	7	5	1	6	7	1	2	0	0	1.663571	4.25298
2.2	13	12	0	22	7	4	1	5	7	2	1	0	0	1.650985	3.93744
2.3	15	8	0	25	8	5	1	5	9	0	1	2	1	1.677898	3.75943
2.4	13	11	0	24	13	4	0	9	6	1	2	0	0	1.635511	4.38930
2.5	11	10	0	25	12	5	1	9	7	0	1	0	0	1.663943	4.88432
2.6	9	16	0	23	17	3	0	10	4	1	1	0	0	1.570649	3.84252
2.7	7	14	0	26	17	5	0	9	4	0	1	0	0	1.592818	4.13916
2.8	8	13	0	26	10	5	1	8	5	0	1	1	1	1.621768	3.83438
2.9	8	13	0	27	12	3	1	10	3	2	1	0	1	1.626602	4.39177
3	10	13	0	28	14	3	0	9	5	0	1	0	0	1.634257	4.62943
3.1	10	14	0	26	13	5	0	9	4	0	1	0	1	1.602805	4.60175
3.2	9	15	0	27	13	5	0	10	4	1	1	0	1	1.60413	3.80093
3.3	9	13	0	29	16	5	0	9	4	0	1	0	1	1.613361	4.88120
3.4	11	13	0	30	13	5	0	10	3	0	1	1	1	1.620331	4.07718
3.5	14	9	0	28	11	5	0	9	3	2	1	0	1	1.660518	4.22941
3.6	12	10	0	30	12	5	1	11	4	0	2	1	0	1.659983	4.18152
3.7	13	12	0	29	13	4	0	11	3	0	2	0	1	1.629991	4.09429
3.8	12	14	1	27	14	4	0	11	3	0	2	0	1	1.594108	3.9168
3.9	10	10	0	25	15	8	0	8	3	1	1	1	0	1.609171	4.66298
4	10	10	0	26	18	4	0	9	4	1	2	1	0	1.612924	4.89110
4.1	9	14	1	26	16	4	0	9	4	1	2	0	0	1.589079	4.66757
4.2	8	12	0	27	15	4	0	9	3	0	2	0	1	1.605804	4.42630
4.3	8	14	0	30	15	5	0	10	4	1	2	0	0	1.619938	5.14990
4.4	10	15	1	25	18	4	2	11	6	0	1	1	0	1.588794	4.24188
4.5	11	14	0	28	17	5	1	12	4	1	0	0	1	1.614085	4.36811
4.6	10	14	0	20	28	6	0	11	5	1	1	0	0	1.539942	4.86174
4.7	11	14	0	26	18	4	0	12	4	1	1	0	0	1.602448	4.32036
4.8	9	12	0	31	18	4	0	11	3	1	1	2	0	1.618498	4.24435
4.9	9	13	0	32	20	6	1	11	6	0	0	1	0	1.631576	4.50839
5	14	8	1	32	18	6	0	12	7	1	0	0	0	1.680584	4.12505
5.1	7	14	0	33	22	7	0	12	6	0	0	0	0	1.626058	4.14669
5.2	7	14	0	34	19	7	0	11	5	1	0	1	0	1.628938	4.26508
5.3	7	16	0	32	20	4	0	11	7	0	0	0	0	1.623266	4.98962
5.4	7	17	0	31	25	5	0	11	5	1	0	0	1	1.579345	4.97658
5.5	6	15	1	31	25	6	0	12	4	1	0	1	1	1.576274	4.84749
5.6	6	16	1	31	26	5	0	12	5	2	0	0	0	1.586218	5.02846
5.7	5	14	1	30	25	5	0	12	3	2	1	0	0	1.582782	4.92612
5.8	7	15	1	31	24	5	0	12	3	2	0	0	1	1.58333	5.69495

5.9	8	16	0	32	23	6	0	11	3	1	0	1	1	1.579419	5.58638
6	8	12	0	31	19	5	0	11	3	3	0	0	0	1.634016	4.09225

4. Deprotonation mechanism

4.1 Reax-FF MD simulations

We also studied the deprotonation process and mechanism in detail at 2500 K. Hydrogen on the carbon atom first moved to the nitrogen atom and then generated H_2. Our calculations confirmed that deprotonation preferentially occurred by desorbing dihydrogen atoms at nitrogen atoms.

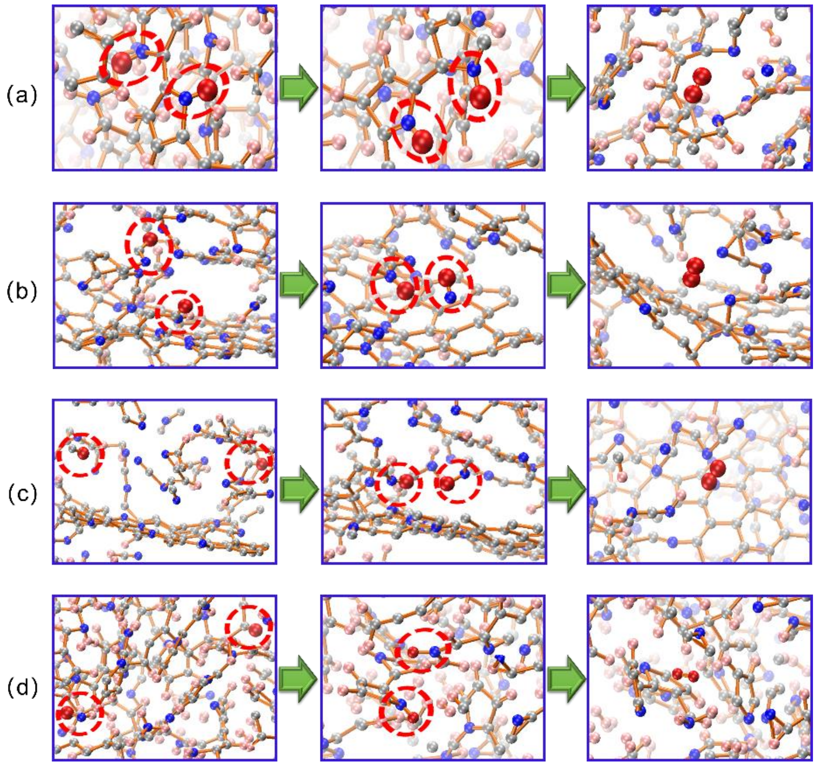

Figure S8. Hydrogen evolution paths at 2500 K.

4.2 NEB calculations

The NEB calculations were applied to explain the dehydrogenation mechanism. The dehydrogenation calculations were performed with saturated tricyclic PPy models.

Dissociation energy of hydrogen atom

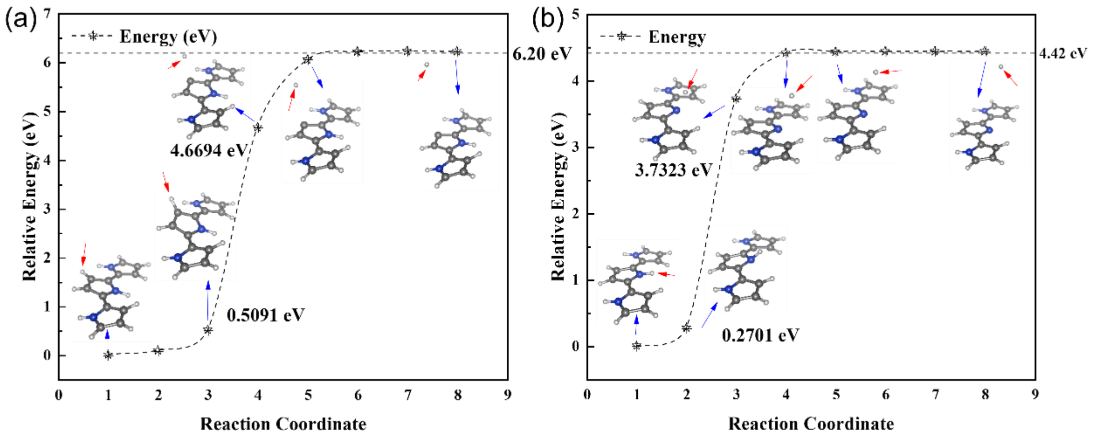

Figure S9. The dissociation energy of hydrogen at the carbon and nitrogen sites, respectively.

As shown in Supplementary Figure S9, using the saturated tricyclic PPy models, we calculated the dissociation energy of hydrogen at the carbon and nitrogen sites, respectively. We found that the dissociation of hydrogen from carbon and nitrogen sites both requires higher energy, and the dissociation energy of a single hydrogen atom from carbon was ~1.8 eV higher than that from nitrogen. To further study the dehydrogenation mechanism during carbonization of PPy, we also calculated the dissociation energy of dihydrogen atoms at carbon and nitrogen sites and the migration activation energy of hydrogen atoms moving on the rings.

C-C Dehydrogenation

As shown in Supplementary Figure S10, the H atom was first desorbed from a C atom, and the H-C bond-breaking created a lower energy barrier (2.4684 eV). Then the H atom bonded with the adjacent C through an energy decreasing process along the ring. The highest energy barrier (4.8173 eV) of C-C dehydrogenation originated from the bond breaking of H_2-C in the final step.

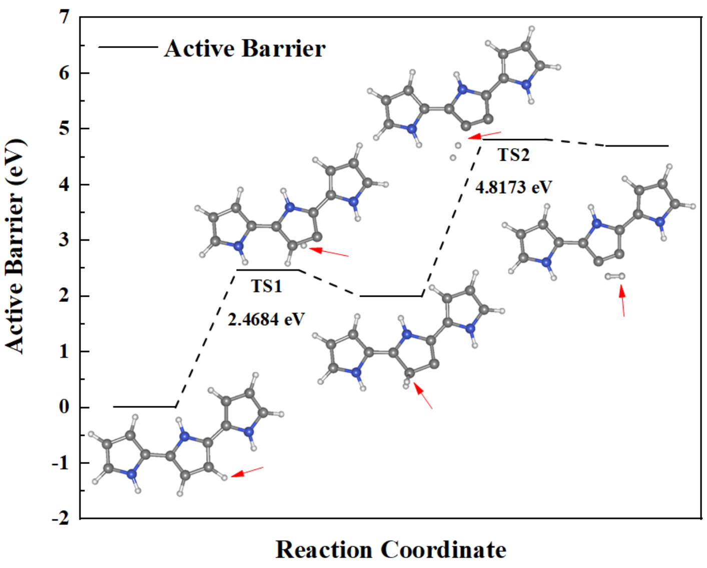

Figure S10. The NEB minimum energy path in the C-C dehydrogenation process (White: H, Gray: C, Blue: N).

N-N Dehydrogenation

Similarly, H on the N atom was also desorbed by transferring on the PPy rings. As shown in Supplementary Figure S11, the H atom first moved from the N atom with a lower N-H bond breaking energy (0.4586 eV). Subsequently, the H atom bonded to the C atom at the other end of the ring through transfer without an energy barrier. The bond breaking of C-H and N-H in the final step created the highest energy barrier (2.9516 eV) for N-N dehydrogenation.

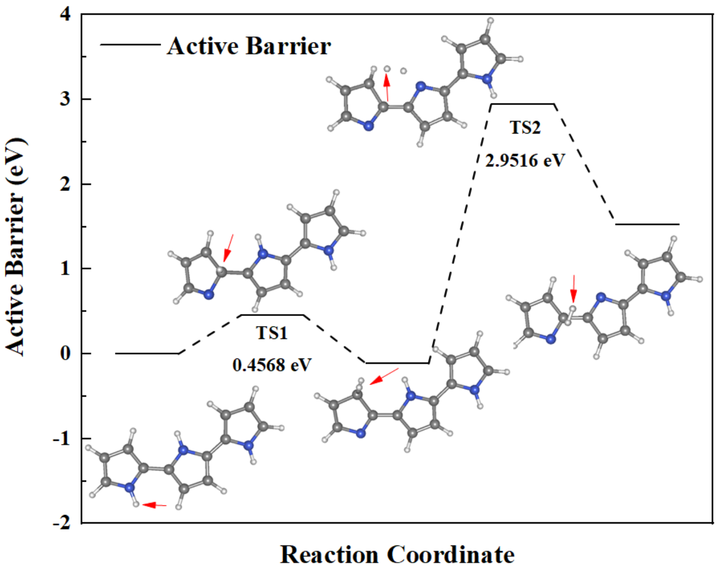

Figure S11. The NEB minimum energy path in the N-N dehydrogenation process (White: H, Gray: C, Blue: N).

C-N hydrogen atom transfer process

MD showed that the H atom on the C atom could be transferred to the N atom after dehydrogenation on the N atom. As shown in Supplementary Figure S12, we calculated the kinetic barrier of H atom transfer from C atom to N atom on the same ring. The H atom was first desorbed from a C atom with a high energy barrier (3.5907 eV), which is much higher than the energy barrier caused by the bond breaking of C-H on saturated PPy. Subsequently, the H atom chemisorbed with the intermediate atom C, and the bond breaking of intermediate C-H created the highest energy barrier in the transfer process (3.9674 eV). The results showed that dehydrogenation becomes more difficult as PPy turns into unsaturated gradually.

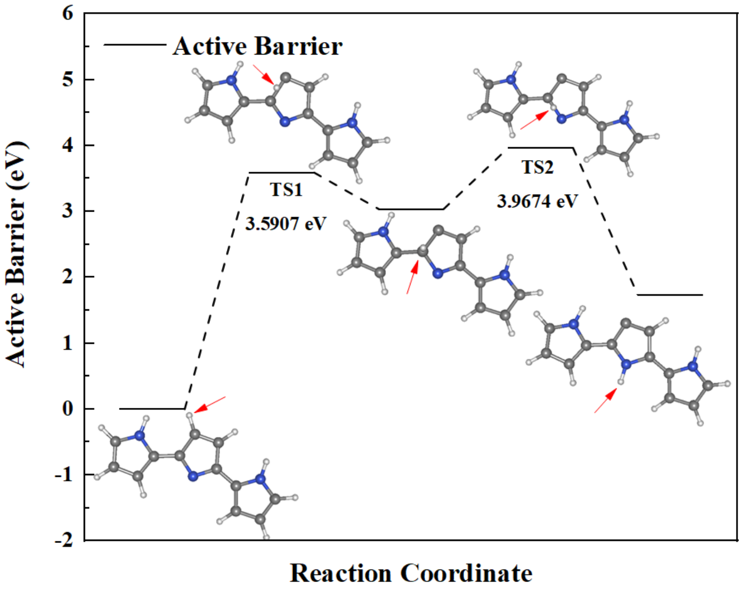

Figure S12. The NEB minimum energy path of the C-N hydrogen atom transfer process (White: H, Gray: C, Blue: N).

As shown in Supplementary Figure S13, the whole process is that the H atoms were first desorbed from C, desorbed from the PPy ring, and transferred to the right. The H-C bond was broken to generate a significant energy barrier (2.5434 eV). The released energy of the combination of H and N atoms led to a downslope of energy. Then two hydrogen atoms were desorbed from N. At this time, there was an energy barrier of (2.8733 eV) relative to the initial structure, and the energy of the final system dropped to the final state. The energy required for dehydrogenation became higher gradually as PPy turned to be unsaturated, requiring a higher temperature for the subsequent dehydrogenation.

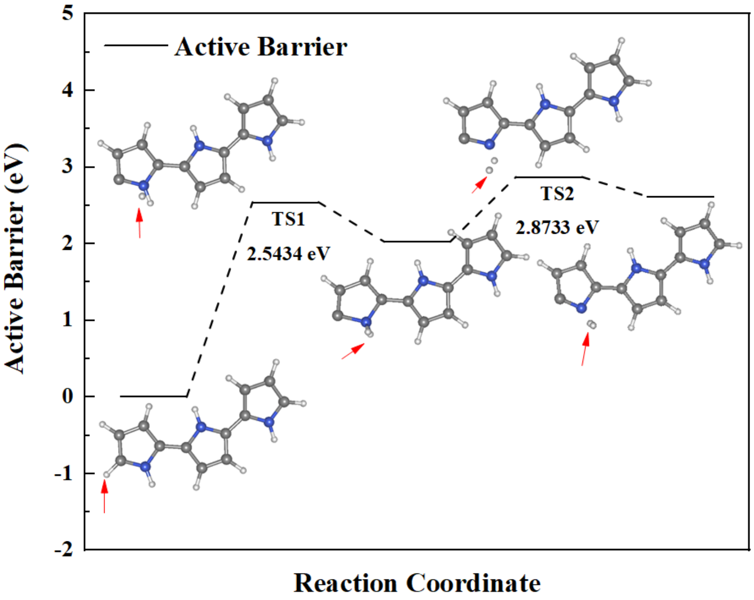

Figure S13. The NEB minimum energy path of the C-N hydrogen atom transfer process (White: H, Gray: C, Blue: N).

Based on the above analysis, we can draw a conclusion that the deprotonation preferentially occurred by desorbing dihydrogen atoms at nitrogen atoms. Furthermore, the C atom can produce hydrogen when the ambient energy is high enough to break the C-H bonds.
